# Shifting the narrative from living at risk to living with risk: validating and pilot-testing a clinical decision support tool: a mixed methods study

**DOI:** 10.1186/s12877-023-04068-w

**Published:** 2023-05-31

**Authors:** Heather MacLeod, Nathalie Veillette, Jennifer Klein, Nathalie Delli-Colli, Mary Egan, Dominique Giroux, Marie-Jeanne Kergoat, Shaen Gingrich, Véronique Provencher

**Affiliations:** 1 Regional Geriatric Program of Eastern Ontario, Ottawa, ON Canada; 2 School of Rehabilitation, Faculty of Medicine, Université de Montréal, Montreal, QC Canada; 3 Institut Universitaire de Gériatrie de Montréal (IUGM) Research Center, Montreal, QC Canada; 4 Glenrose Rehabilitation Hospital, Edmonton, AB Canada; 5 School of Social Work, Faculty of Arts, Humanities and Social Sciences, Université de Sherbrooke, Sherbrooke, QC Canada; 6 Research Centre on Aging, Sherbrooke, QC Canada; 7 School of Rehabilitation Sciences, Faculty of Health Sciences, University of Ottawa, Ottawa, ON Canada; 8 Department of Rehabilitation, Faculty of Medicine, Université Laval, Québec City, QC Canada; 9 Centre of Excellence on Aging, Québec, QC Canada; 10 Department of Medicine, Faculty of Medicine, Université de Montréal, Montreal, QC Canada; 11 North East Specialized Geriatric Centre, Sudbury, ON Canada; 12 School of Rehabilitation – Pavillon Gérald-Lasalle, Faculty of Medicine and Health Sciences, Université de Sherbrooke, 3001 12e Avenue Nord, Sherbrooke, QC J1H 5N4 Canada

**Keywords:** Risk assessment, Risk management, Risk-taking, Clinical decision-making, Older adult, Delphi, Validation

## Abstract

**Background:**

When there are safety concerns, healthcare professionals (HCPs) may disregard older adults’ wishes to return or remain at home. A paradigm shift is needed for HCPs to move from labelling older adults as living at risk to helping them live with risk. The Living with Risk: Decision Support Tool (LwR:DST) was developed to support older adults and HCPs with difficult decision-making regarding living with risk. The study objectives were to: (1) validate, and (2) pilot-test the LwR:DST in hospital and community settings.

**Methods:**

The study was conducted across Canada during the pandemic. The LwR:DST’s content was validated with quantitative and qualitative data by: (1) 71 HCPs from hospital and community settings using the Delphi method, and (2) 17 older adults and caregivers using focus groups. HCPs provided feedback on the LwR:DST’s content, format and instruction manual while older adults provided feedback on the LwR:DST’s communication step. The revised LwR:DST was pilot-tested by 14 HCPs in one hospital and one community setting, and 17 older adults and caregivers described their experience of HCPs using this approach with them. Descriptive and thematic analysis were performed.

**Results:**

The LwR:DST underwent two iterations incorporating qualitative and quantitative data provided by HCPs, older adults and caregivers. The quantitative Delphi method data validated the content and the process of the LwR:DST, while the qualitative data provided practical improvements. The pilot-testing results suggest that using the LwR:DST broadens HCPs’ clinical thinking, structures their decision-making, improves their communication and increases their competence and comfort with risk assessment and management. Our findings also suggest that the LwR:DST improves older adults’ healthcare experience by feeling heard, understood and involved.

**Conclusions:**

This revised LwR:DST should help HCPs systematically identify frail older adults’ risks when they remain at or return home and find acceptable ways to mitigate these risks. The LwR:DST induces a paradigm shift by acknowledging that risks are inherent in everyday living and that risk-taking has positive and negative consequences. The challenges involved in integrating the LwR:DST into practice, i.e., when, how and with whom to use it, will be addressed in future research.

**Supplementary Information:**

The online version contains supplementary material available at 10.1186/s12877-023-04068-w.

## Background

Most older adults wish to remain at home as they age [1, 2]. This presents a dilemma for healthcare professionals (HCPs) when older adults experience changes in health or social support that seem to put them at risk in their own homes. There is a natural tension between respecting older adults’ decisional autonomy concerning living at home and urging them to move to an assisted living environment to avoid anticipated harm. In other words, HCPs struggle to balance the bioethical principles of autonomy, beneficence, and non-maleficence [3]. This dilemma may cause moral distress for HCPs when respecting the patient’s wishes does not match their personal values. This may explain why, during discharge planning, HCPs tend to prioritize their judgment of what is required for safety over their patients’ preferences [4].

This clinical dilemma is exacerbated by the way HCPs understand and approach risk. HCPs tend to define risk negatively [5], prioritize the negative consequences [5], and focus on the physical consequences [6]. Furthermore, HCPs have different definitions of living at risk [7]. Current risk assessments often focus narrowly on specific safety issues like falls [8] or generate a decontextualized risk score [9–11]. They often fail to consider patients’ perceptions or preferences regarding strategies to mitigate risk, consider older adult and caregiver risk tolerance [7, 12] or consider how important risky activities are for the person. This can lead to generic recommendations that do not address older adults’ concerns or the prescription of types of support that are unsustainable or unacceptable to patients and caregivers [4, 13]. Moreover, current methods of pre-discharge risk assessment are limited. For instance, when older adults are hospitalized, risk assessment accuracy may be compromised by unfamiliarity with the hospital environment [14], cognitive fluctuations due to medication or fatigue, lack of information regarding home hazards [15] and difficulty predicting clinical progression. To our knowledge, only one patient-centered tool has been developed to support risk assessment in people with dementia [16]; however, it is not designed for use in the hospital discharge process. Instead, it focuses on activities viewed as risky by patients and caregivers and does not address the ethical dilemmas that result from the inherent trade-offs between safety and autonomy and the conflicting views of patients, caregivers and HCPs [16].

**Fig. 1:**
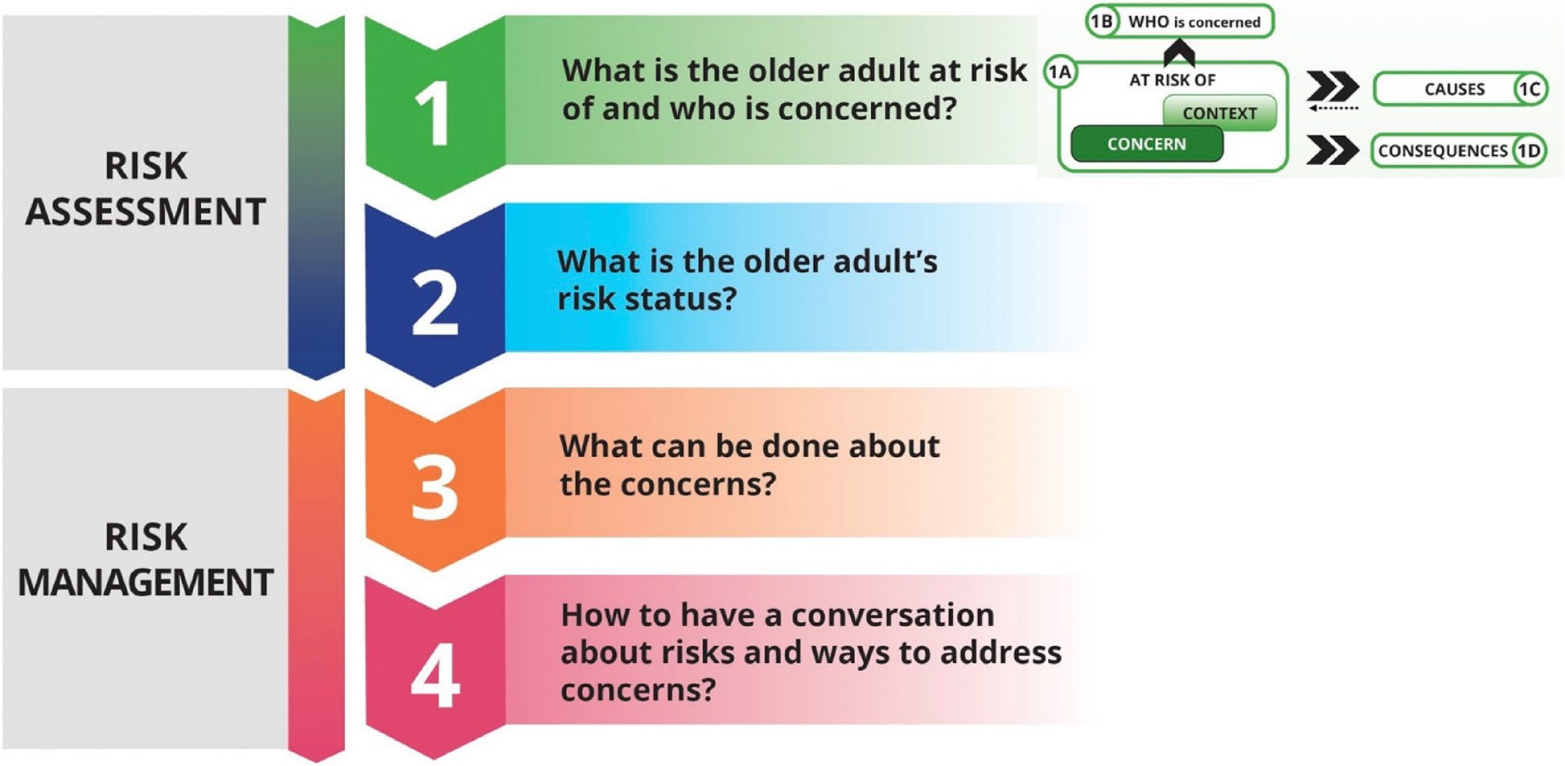
The living with risk: decision support tool (version 2.2)

HCPs are better able to support patient safety and autonomy when they broaden and clarify their definitions of risk and living at risk, and when they balance their approach to risk assessment and management [7]. This balanced approach acknowledges that risk: (1) is inherent in everyday living, (2) has both emotional and physical consequences [6], and (3) can produce both negative and positive consequences [17]. To support this broadened approach, the Living with Risk: Decision Support Tool (LwR:DST) was developed from best risk assessment and management practices in the community [7] and recently adapted to the hospital environment. The LwR:DST (Fig. 1) consists of four steps that determine what the older adult is at risk of and who is concerned, ascertains the older adult’s risk status, establishes what can be done about the concerns, and supports conversations with the older adult, caregiver and team members about the risks and ways to address the concerns. The LwR:DST is innovative as it incorporates a balanced approach to risk assessment by: (1) recognizing that risks have negative consequences such as rehospitalization and positive ones such as increased resilience and quality of life [18]; (2) looking at living with risk along a continuum from safe to unsafe rather than in the traditional dichotomous way of safety versus autonomy; (3) explicitly considering the likelihood, frequency, severity, complexity and imminence of the harm, and factors that compound or mitigate the risks identified; and (4) balancing what can be done to reduce harmful events and negative consequences while at the same time incorporating the older adult’s and their caregiver’s wishes, values and beliefs. However, this tool has not yet been tested for validity or practicality in healthcare settings.

The overall goal of this study was to refine the LwR:DST to ensure that it is a valid and practical risk assessment method that more broadly considers risk in hospitalized older adults preparing for discharge. More specifically, the two study objectives were to: 1) validate the LwR:DST’s content in community and hospital settings, and (2) pilot-test the LwR:DST to verify that it facilitates the complex risk assessment of older adults living with frailty for both hospital- and community-based HCPs.

## Methods

**Fig. 2:**
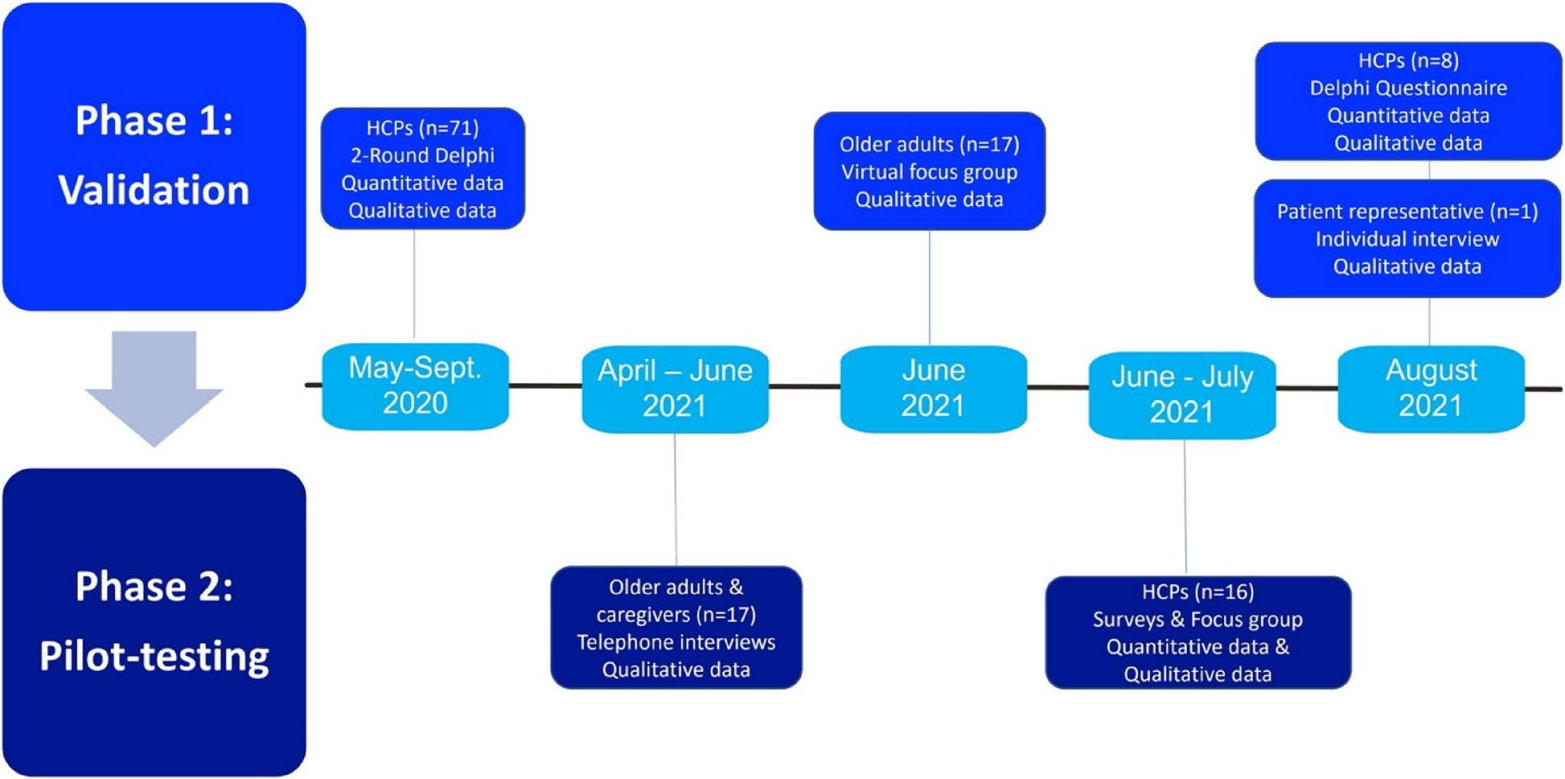
Study phases summary

This convergent mixed-method two-phase study (Fig. 2) utilized an integrated knowledge translation (KT) approach [19] as Canadian knowledge users (HCPs, older adults and caregivers) were involved in both phases. The data collected in each phase were incorporated into the next phase. Therefore, the LwR:DST’s content and format, instruction manual and accompanying worksheets were adapted throughout the two phases based on input from the knowledge user participants. Each study phase is described below. As illustrated in Fig. 2, some elements of Phase 1 overlapped with Phase 2 due to COVID-19 related restrictions around recruitment of older adults and French-speaking HCPs. Recruitment resumed when these restrictions were lifted. All questions in the Delphi questionnaires (Phase 1), focus groups (Phases 1 and 2), surveys (Phase 2) and interviews (Phase 2) were pretested with the relevant population (HCPs, older adults and/or caregivers) and changes were made to ensure the questions were clear.

### Pre-study phase: adapting the LwR:DST (Version 1.0) to the hospital context

Prior to the start of the study, the LwR:DST was adapted to the hospital context with one workshop in January 2019 (Alberta, Canada) and another in November 2019 (Québec, Canada) for a total convenience sample of 81 HCPs, 1 older adult, 6 caregivers and 6 researchers, all familiar with the inpatient hospital clinical setting. These participants were asked to: (1) describe how the LwR:DST differed from current practice, (2) list the individual and/or organizational obstacles or limitations in using the LwR:DST in a hospital setting, (3) highlight the benefits of using the LwR:DST, and (4) provide recommendations to improve the LwR:DST’s format and content (refer to Additional file 7 for questions). The participants requested the development of worksheets, and their input was incorporated into the LwR:DST and accompanying instruction manual, resulting in Version 2.0 which was used for Phase 1 of this study.

### Phase 1: validation of the LwR:DST (Version 2.0) by HCPs

To enhance the LwR:DST’s content validity, hospital- and community-based HCPs representing a variety of experience levels, practice settings and disciplines were recruited to participate in a Delphi process. The Delphi process was chosen as it is a method recommended to achieve consensus among a group of experts over two to four rounds of questions [20] and it has previously been used to validate content [21]. Participants were recruited via email from the research team’s professional contacts (and their contacts) in geriatric settings across five provinces in Canada. Inclusion criteria included any HCP working with community-dwelling older adults with complex needs. Both qualitative and quantitative data were collected during the Delphi process (refer to Additional file 7 for questions). The HCPs were asked to provide feedback on the LwR:DST’s content, format and instruction manual with respect to their usefulness and content adequacy in line with best practices. Consensus for the quantitative data was pre-set at 75% [22] and this was achieved in two rounds. The Delphi participants had two weeks to complete each round of questions and at least three weeks between each round. One reminder email was sent midway through each round. The targeted sample size was 45 HCPs and 79 were recruited.

### Phase 1: validation of the LwR:DST (Version 2.2) by older adults

A convenience sample of 17 older adults was recruited through the research team’s contacts for a 60-minute virtual semi-structured focus group. To be included, participants needed to be 65 years or older and have the resources to participate in a virtual focus group. The older adults were asked to provide input on Step 4 of the LwR:DST: ‘How to have a conversation about risks and ways to address concerns?’. More specifically, the participants were asked: (1) what they wanted to know in these conversations, (2) how they wanted to have these conversations, and (3) what was important to them in these conversations (refer to Additional file 7 for questions). Prior to the focus group, a patient representative reviewed and provided feedback on the focus group questions. The focus group was recorded, transcribed in full and inputted into NVivo (version 12).

### Phase 2: pilot-testing the LwR:DST (Version 2.2) with healthcare professionals, older adults and caregivers

#### Pre-use of the LwR:DST during usual care

Two community and two hospital clinical settings providing healthcare to people 65 years or older were recruited through the research team’s contacts to pilot-test the feasibility of using the LwR:DST in real-life contexts. HCPs who consented to participate needed to use the LwR:DST during usual care for a period of 8 weeks. All HCPs in the participating programs at each clinical setting were offered a 60-minute online training session and asked to answer pre- and post-training questions (refer to Additional file 7 for questions). Training included a primer on risk assessment as well as an overview of the LwR:DST and research protocol. Adapting innovations to HCPs’ clinical context requires an understanding of the current situation in order to determine the gap between practice and the innovation (i.e., LwR:DST) [23]. Therefore, the pre-training online questionnaire contained: (1) three qualitative questions aimed at understanding participants’ current practice and challenges pertaining to risk assessment and management, and (2) two 10-point Likert questions asking HCPs about their confidence in their ability to have conversations about care and/or discharge with their complex patients and how informed they feel regarding assessing risk. In the post-training questionnaire, participants were asked the same two questions as well as to assess the degree to which training provided resources and strategies to enhance their approach to risk assessment and management. Using the CPD-Reaction tool [24], they were also asked about their intention to use the LwR:DST following training. Higher scores for each item indicate higher levels of intention and thus a greater likelihood of adoption since intention is considered the most proximal cause of a change in behavior [25]. Following training, HCPs were instructed to use the LwR:DST for two months as part of usual care.

#### Post-use of the LwR:DST during usual care

Older adults and their caregivers who had the LwR:DST used with them were asked to take part in individual semi-structured 30-minute telephone interviews regarding their satisfaction with the risk conversations and perceptions of safety and autonomy at home (refer to Additional file 7 for questions). Older adult participants needed to be 65 years or older and they had to be able to participate in the interview in English or French, as did their caregivers. Research assistants with substantial experience in geriatric care and qualitative interviewing conducted the phone interviews two weeks post-hospital discharge or two weeks post-LwR:DST use in the community to ensure recall of the care experience. At the end of the two months of use of the LwR:DST in usual care, the HCPs were asked to participate in a 60-minute semi-structured virtual focus group to provide their perspective on the usefulness of the tool, its fit with their type of patients, suggestions for improvements, and obstacles to use (refer to Additional file 7 for questions). The interviews and focus group were recorded, transcribed in full and inputted into NVivo (version 12).

### Data analysis

#### Mixed methods

This study design used qualitative data supplemented with quantitative data. All qualitative data (as indicated in Fig. 2) were analyzed for themes by author (HM) using the procedure described by Braun and Clarke [26]. The themes generated were semantic in nature [26] to keep them close to the participants’ perspective and ensure clinical relevance, relatability, and usefulness. Qualitative categories and themes from Phase 1(HCPs) were reviewed by author (VP) to ensure they accurately reflected the codes and participants’ viewpoints. Phases 1(older adults) and 2 qualitative transcripts were co-coded independently by HM and a research assistant. They then reviewed their codes, categories and themes and discussed any differing analyses until consensus was obtained. Qualitative data rigor was obtained by first ensuring that the transcripts were accurate, that themes emerged from the data via coding and then categories, which were reviewed and discussed, and consensus obtained with at least two researchers. The data were triangulated between qualitative and quantitative data (Phase 1) and across Phases (1 and 2).

The quantitative data were analyzed using descriptive statistics with means and percentages and a paired t-test (parametric) or Wilcoxon signed-rank test (non-parametric) was used to determine statistical significance between the pre- and post-training online sessions. In Phase 1(HCPs), the qualitative data added context to the quantitative data and in the remaining phases 1(older adults) and 2 (older adults, caregivers and HCPs) the quantitative data provided context (i.e., demographic information) for the qualitative data.

## Results

**Fig. 3:**
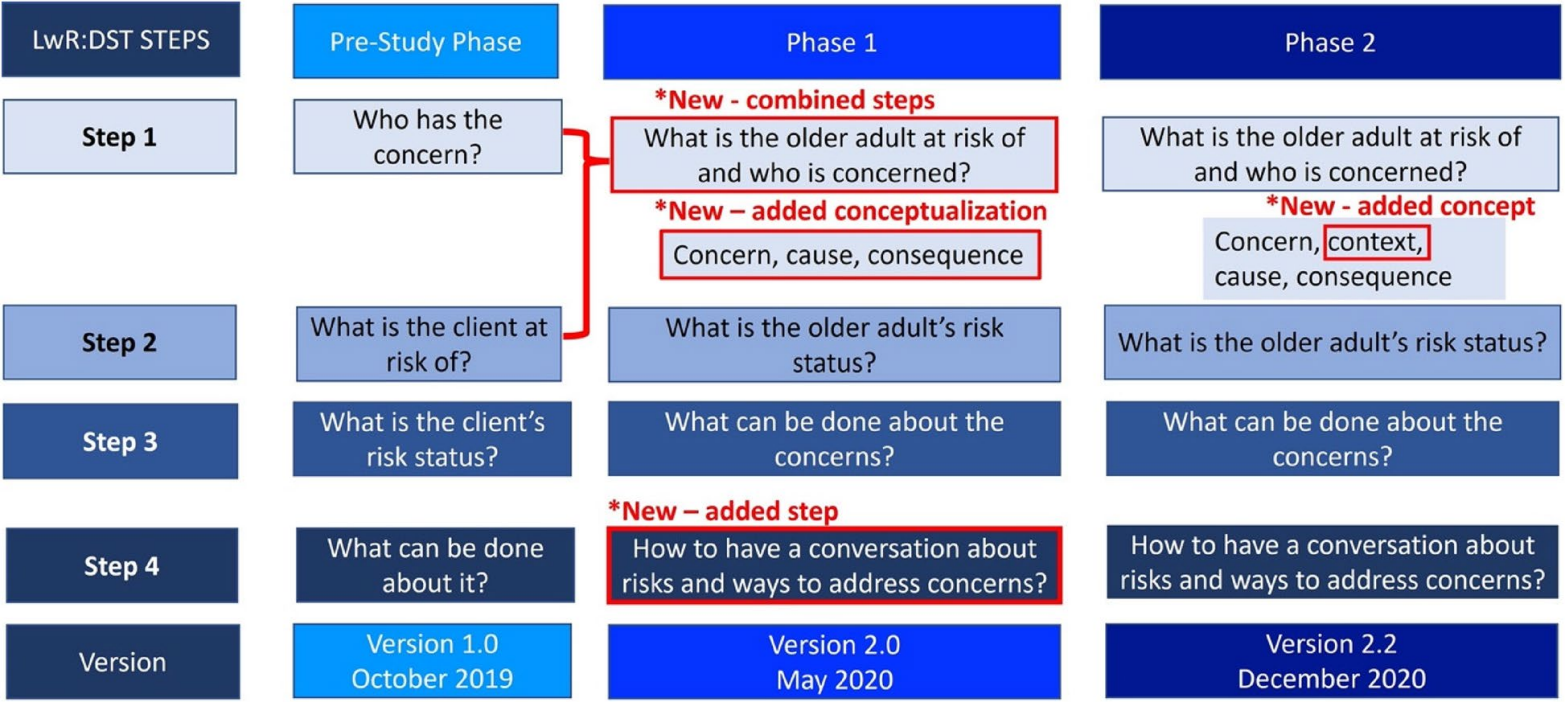
Iterative incorporation of participant input by study phase for each LwR:DST step

**Fig. 4:**
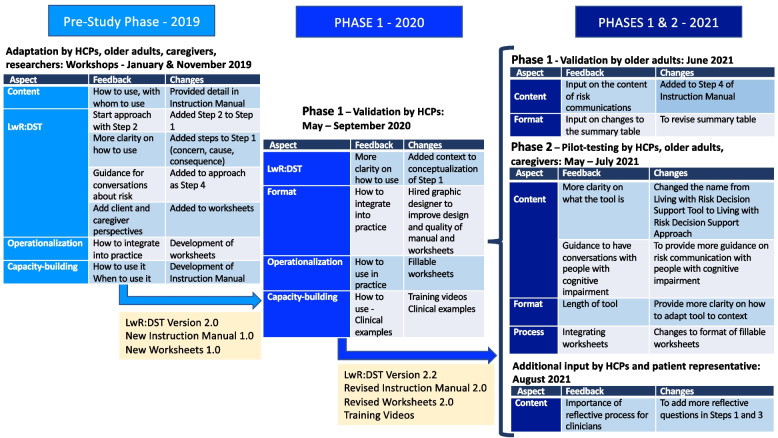
Evolution of the LwR:DST through the study phases based on participant feedback

Key stakeholders (older adults, caregivers, HCPs, patient representatives and researchers) provided input concerning the LwR:DST throughout the study’s two phases of validation and pilot-testing. Figure 3 depicts what changes were made to the steps of the LwR:DST after each phase (including the pre-study phase). Figure 4 highlights the evolution of the LwR:DST and the accompanying tools through the study phases based on participant input.

### Phase 1: validation of the LwR:DST by HCPs

Seventy-nine and 77 English and French-speaking HCPs from across Canada working in either a hospital or the community were sent the Round 1 and the Round 2 questions respectively, using the Delphi process. Seventy-one HCPs completed Round 1 for a response rate of 90% while 49 participants completed Round 2 for a response rate of 64%. Table 1 shows the Delphi participants’ clinical setting, years of experience, discipline and language of the LwR:DST that they reviewed. The predetermined threshold of 75% (for a cut-off score of 7 on the Likert scale) was reached for all questions except one in Round 1 and for all questions in Round 2, which eliminated the need for any more rounds. An additional 7 HCPs and 1 patient representative completed the Delphi questionnaires in August 2021. This aimed to increase the number of participants providing feedback on the French version of the LwR:DST as recruitment of French-speaking HCPs from a particular region of Canada during the original Delphi timeframe was prohibited due to COVID-19 related burden on the healthcare system.

**Table 1:** Delphi: demographic information from participants validating the LwR:DST

	May – September 2020 (*n* = 71)	August 2021 (*n* = 8)
**Clinical setting**		
Hospital	56%	62%
Community	41%	38%
Other	3%	--
**Years of experience**		
11+	66%	75%
26+	27%	38%
**Discipline**		
Occupation therapist	50%	62.5%
Registered nurse	17%	12.5%
Social worker	13%	0%
Physiotherapist	7%	0%
Medical doctor	7%	12.5%
Other	6%	12.5%
**Language of LwR:DST**		
English	81%	--
French	19%	100%

#### Delphi quantitative and qualitative results

As indicated in Table 1, Delphi questionnaire participants were almost equally divided between hospital and community settings. Two-thirds of the participants had 11 or more years of experience. Although participants were from a variety of disciplines, occupational therapists, who traditionally assess older adults’ home safety [27], accounted for 50%. Most respondents reviewed the English version of the tool. As indicated in Table 2, only one question concerning the format of the LwR:DST (to what extent is the format of the tool ‘user-friendly’) did not achieve a consensus over 75%. Seventy-seven participants were emailed the Round 2 questionnaires related to format and a consensus of over 75% was achieved (cut-off score of 7 on the Likert scale) for using colors in the safety continuum and for having fillable worksheets to operationalize use of the LwR:DST (Table 2).

**Table 2:** Delphi results – round 1 and 2: quantitative data validating the LwR:DST

Questions^a^	% ≥ 7^b^
**Round 1**	
To what extent do you think that the tool is **‘useful’** to your work in assessing and managing risks that older adults may face?	86
To what extent do you think that each step of the tool is **‘useful’** to your work in assessing and managing risks that older adults may face?	
Step 1: What is the older adult at risk of and who is concerned?	98
Step 2: What is the older adult’s risk status?	87
Step 3: What can be done about the concerns?	89
Step 4: How to have a conversation about risks and ways to address concerns?	85
Does the 4-step approach contain **‘adequate’** content to be able to assess and manage the risks associated with older adults?	83
To what extent do you think that each step of the tool contains **‘adequate’** content to be able to assess and manage the risks associated with older adults?	
Step 1: What is the older adult at risk of and who is concerned?	89
Step 2: What is the older adult’s risk status?	86
Step 3: What can be done about the concerns?	85
Step 4: How to have a conversation about risks and ways to address concerns?	85
For Step 1, does the list represent the concerns you have about your older adult patient? (Step 1: What is the older adult at risk of and who is concerned?) - YES	89
To what extent is the format of the tool **‘user-friendly’**?	69
To what extent is the information in the instruction manual **‘sufficient’** to be able to use the tool?	83
To what extent is the format of the information in the instruction manual **‘user-friendly’**?	80
**Round 2**	
To what extent do you **‘agree’** that it is clinically useful for the safety continuum to include colors?	82
As we make changes to the format based on your feedback from Delphi 1, to what extent do you **‘agree’** that it is clinically useful to have a worksheet that can be filled out?	86

^a^Likert questions: 1-3 (not ‘X’); 4-6 (somewhat ‘X’); 7-9 (‘X’) where X = ‘useful’, ‘adequate’, ‘user-friendly’, ‘sufficient’ or ‘agree’

^b^Consensus achieved if at least 75% of the respondents answered 7 or over on the Likert questions

The qualitative data revealed that the HCPs found that the LwR:DST and each of the 4 steps were useful in assessing and managing risks in older adults, and that the content of both was adequate. The HCPs also found that the format of the instruction manual was user-friendly and that it contained sufficient information to be able to use the LwR:DST in practice. The qualitative data indicated that the participants found the LwR:DST to be comprehensive, systematic and patient-centered, as well as useful for clinical reasoning, communication, and documentation. For instance, a hospital-based clinician provided the following reflection on the benefits of the LwR:DST:

*“I think that it [LwR:DST] helps to organize ideas and concerns and facilitates collaborative problem-solving around risk. In my experience, these conversations are already happening but not in a way that the whole team is always involved and there is a clear action plan. I think the tool and the worksheets help to consolidate information and, in the end, save time for everyone because there isn’t so much back-and-forth (hospital HCP participant)*."

The participants suggested that the format of the instruction manual and clinical examples could be improved, and they requested more information in the instruction manual on: (1) when, how and with whom to use the LwR:DST, and (2) tips for communicating risk with older adults, caregivers and, where applicable, their team. They also acknowledged that time would likely be an overall barrier to adopting the LwR:DST in practice. More specifically, the participants referred to the lack of time available to learn and adopt a new tool in practice, the time needed to engage with the team, patient and/or their caregiver when using the tool, and the time needed to involve the team in the risk assessment and management process. The following community-based participant described the difficulty of integrating the LwR:DST into practice:

*“Having the time in my practice to apply it. Although I can already see how great it would be to apply it, with the ongoing demands of the job and the number of patients we are expected to see, it may be difficult to add it to our schedules (community HCP participant)*."

See Additional file 1 for more quotations from participants that reflected all these themes.

### Phase 1: validation of the LwR:DST by older adults

Seventeen older adults participated in one of three 60-minute virtual focus groups. The majority (82%) of participants were female and the average age was 75. The older adults answered the questions about conversations about risk and ways to address safety concerns from the perspective of both an older adult and a caregiver, as all had been caregivers to an older adult. The participants wanted the content of risk conversations to include information about the older adult’s condition, HCPs’ impressions, older adult’s options and the rationales behind HCPs’ decisions. The participants indicated that they wanted (a) these conversations to be collaborative with the right people, (b) to be invited to provide solutions, (c) to know their options, and (d) quality of life to be prioritized over safety. The participants differed in who they felt should be making the decisions. When speaking as an older adult patient, participants wanted the older adult to make the decisions but when speaking as a caregiver, they wanted HCPs to make the decisions. The following quote highlights the participant’s preference for quality over safety:

*"I’m going to step back into my previous life of having a 98-year-old mother living with me since she was 96 and I really believe that it’s important to capture risk versus quality of life, and I really think we need to understand because as these people age and live to be older and older and lose people, they know they’re at risk. So maybe the issue becomes the quality and I think that’s paramount in helping them to make decisions, too, because if they – I think back to our situation and staying at home was paramount. That was what mattered, no matter what (older adult participant)."*

The quotations in Additional file 2 reflect all the themes discussed above.

### Phase 2: pilot-testing the LwR:DST with HCPs, older adults and caregivers

#### Pre-use data - HCPs

Attempts were made to recruit one community program and one hospital program for both English- and French-speaking clinical settings for a total of four sites to pilot-test the feasibility of using the LwR:DST in practice. Both French-speaking settings (one community and one hospital) were unable to participate due to competing priorities related to the COVID-19 pandemic. The remaining two English teams consisted of an urban community team of nurses and community health workers serving isolated older adults and an inpatient geriatric rehabilitation unit in a large urban rehabilitation hospital.

Nineteen HCP participants (10 community and 9 hospital) completed the pre-training questions, and 16 HCPs completed the post-training questions. Table 3 details the demographic information of the participants who completed the training. The training resulted in a statistically significant improvement in the HCPs feeling more informed on how to assess risk after the training. However, the improvement in post-training confidence level of their ability to have risk conversations with their complex patients was not statistically significant (see Table 4 for details).

**Table 3:** Phase 2 pre-/post-training: participant demographic information

	Site					
	Hospital		Community		Total	
	Pre (*n* = 9/39)	Post (*n* = 8/39)	Pre (*n* = 10/11)	Post (*n* = 8/11)	Pre (*n* = 19)	Post (*n* = 16)
**Discipline**						
RN	2	2	3	2	5	4
OT	5	5			5	5
PT	2	1			2	1
CHW			5	3	5	3
Other			1	2	1	2
Management			1	1	1	1
**Years of experience**						
Average (years)	18		7.6			
Range (years)	4-39		0-30			

*RN* Registered nurse, *OT* Occupational therapist, *PT* Physiotherapist, *CHW* Community health worker

**Table 4:** Pre-/post-training quantitative information

Question	Time	Paired samples statistics (*n* = 16)		
		Mean	Standard deviation	Standard error of the mean
Feel confident in my ability to have conversations about discharge and/or care planning with my complex patients (1 = Strongly disagree − 10 = Strongly agree)	Pre	8.21	1.289	0.322
	Post	8.50	0.966	0.242
Feel informed regarding how to assess risk for my patients (1 = Strongly disagree − 10 = Strongly agree)	Pre	7.26	1.401	0.350
	Post	8.30^a^	1.448	0.362

^a^Wilcoxon signed-rank test: *p* = 0.033

While the responses from participants (Additional file 3) as a whole show a comprehensive approach to their pre LwR:DST use practice to risk assessment and management, they suggest some inconsistencies in what the respondents individually considered risk and how they assessed it. The participants’ pre LwR:DST use approach to risk management also varied, although most highlighted the importance of communication in the risk assessment and management process. There was no consensus regarding the challenges associated with risk management, especially when the patient had cognitive impairment affecting insight, or regarding concerns and challenges related to assessing risk in hospital, especially with COVID-related restrictions (e.g., difficulty predicting function at home, limited access to families, unable to trial a weekend at home). The pre-use data suggested that the participants felt they had the knowledge, capabilities, and a high level of intention (Table 5) to use the LwR:DST in usual care but did feel quite confident in having conversations about risk (see Table 4 for details). The current situation and challenges described by the participants suggested that the LwR:DST differed from current practice and could help broaden and systematize the participants’ current approach to risk assessment and management, especially in situations where there is a difference of opinion.

**Table 5:** Intention to use the LwR:DST (2021)

CPD Reaction Tool^24^ questions – 10-point Likert Scale (if not specified: 1- Strongly Disagree to 10- Strongly Agree)	Setting	
	Hospital *n* = 8 (21%) Mean (/10)	Community *n* = 8 (73%) Mean (/10)
1. I intend to use the *Living with Risk: Decision Support Tool (LwR:DST)* to assess and mitigate a patient’s risk.	8.4	8.3
2. To the best of my knowledge, the percentage of my colleagues who will use the *LwR:DST* is ____%.	78%	83%
3. I am confident that I could use the *LwR:DST* to assess and mitigate a patient’s risk if I wanted to.	8.1	8.9
4. Using the *LwR:DST* to assess and mitigate a patient’s risk is the ethical thing to do.	7.8	8.5
5. For me, using the *LwR:DST* to assess and mitigate a patient’s risk would be: (1- Extremely Difficult to 10- Easy)	7.3	7.3
6. Now think about a co-worker whom you respect as a professional. In your opinion, will he/she use the *LwR:DST* to assess and mitigate a patient’s risk?	7.0	7.9
7. I plan to use the *LwR:DST* to assess and mitigate a patient’s risk.	8.4	8.3
8. Overall, I think that for me, using the *LwR:DST* to assess and mitigate a patient’s risk would be: (1- Useless to 10- Useful)	7.7	8.8
9. Most people important to me in my profession will use the *LwR:DST* to assess and mitigate a patient’s risk.	7.7	7.5
10. It is acceptable to use the *LwR:DST* to assess and mitigate a patient’s risk.	8.8	8.8
11. I have the ability to use the *LwR:DST* to assess and mitigate a patient’s risk.	8.6	9.0
12. Overall, I think that for me, using the *LwR:DST* to assess and mitigate a patient’s risk would be: (1- Harmful to 10- Beneficial)	8.4	9.0

#### Post-use qualitative focus group themes - HCPs

Fourteen HCPs (8 community and 6 hospital) participated in one of four virtual 60-minute focus groups. The HCPs thought the tool was useful for improving clinical thinking, communication, HCP and patient outcomes, and the clinical care process. They felt that the LwR:DST fit well with their patients and was useful when patient, caregiver and HCPs had different perspectives. Participants agreed with the improvements made to the worksheets (fillable PDF) but requested further improvements in the format (length of tool), content (what the tool is), and process related to using the tool (how to integrate into practice and how to use with patients with cognitive impairments). The participants suggested that the obstacle to using the LwR:DST centered around how to integrate it into their own clinical context and processes. The subjectivity of the risk analysis was mentioned as an obstacle to use as some participants still wanted a more objective process (i.e., a predictive tool). Despite suggestions for improvements and identified obstacles to use, participants reported a positive impact on both the older adult and the HCPs when the LwR:DST was used. They also indicated that the LwR:DST facilitated improved conversations with the older adult and increased the HCP’s confidence. The following quote acknowledges the time required to integrate new techniques into practice but also highlights the benefit of using the LwR:DST:

*“It was really—at the beginning, to be honest, it was more work for me, but when I chose two of my clients, it was helpful, really. I was surprised because at the beginning, to be honest, [my reaction] was, oh no, oh man. But after working with the client, and my client, one of them specifically, she was pleased with the questionnaire and everything. Yeah, and we’re still talking about that. I think it was a great tool.” (community HCP participant).*

Additional file 4 shows more quotes relating to all themes discussed above.

#### Post-use data – older adults and caregivers

A total of 17 older adults and/or caregivers (10 from the community setting and 7 from the hospital setting) participated in 30-minute telephone interviews while 16 declined for a variety of reasons, such as feeling overwhelmed or too ill to participate, or confused about the consent form. More caregivers from the hospital setting participated in the telephone interviews (5/7 interviews) because some older adults were too ill to participate, while more older adults from the community setting participated in the telephone interviews (8/10 interviews) since many did not have caregivers. Finally, there was a majority of males from the hospital setting (5/7), while there were a few more females in the community setting (6/10).

Additional file 5 summarizes the participants’ themes related to their satisfaction with the care conversations and their perceptions of the older adults’ safety and autonomy at home. Both older adults and caregivers were satisfied with the care conversations since they felt heard and engaged in the process, as described in the following quote:

*“I thought they handled things pretty fairly. They listened to what I had to say, and I didn’t like—I didn’t want them to boss me around, so to speak. They listened to me, and they cooperated with me and so in that respect, I was quite satisfied.” (hospital older adult participant)*.

The participants described their content needs for the care conversations. In addition to understanding the services being arranged or available, they also wanted to understand the HCPs’ concerns and to be provided with the rationales for their decisions. The caregivers also needed to understand the requirements of their role as caregivers. For instance, the caregiver participants from the hospital setting noted a need to be an advocate and have medical expertise that they did not possess. Process-related themes centered around difficulties related to transitioning home and therefore to other organizations and the process of who makes the decisions. For example, caregiver participants from the hospital setting felt abandoned by the hospital and did not know who to contact when the older adult’s situation deteriorated after the return home. Lastly, older adult participants expressed the importance and benefits of having a positive therapeutic relationship, where they were understood and instilled with hope and positivity.

## Discussion

A systematic and balanced process to guide HCPs’ decision-making in assessing and managing risks in the clinical context of older adults with complex needs is currently lacking. A clinical decision support tool (LwR:DST) developed in the community supports a broader approach to risk assessment within a shared decision-making framework but its content and use required scientific validation. Shorter hospital stays [28] and the risk of adverse outcomes post-hospitalization [29] pointed to the need to use the LwR:DST as part of the hospital discharge planning process. For these reasons, this study used an integrated KT approach to validate then pilot-test the LwR:DST in hospital and community settings to support the transition in care and the management of risks at home.

Our findings suggest that the LwR:DST’s validated content and process now support the paradigm shift needed for HCPs to acknowledge that risk is inherent in everyday living, has both positive and negative outcomes, has physical, emotional and social consequences, encourages the leveraging of older adults’ strengths, and requires the participation of older adults and their caregivers in the decision-making process.

Our findings from the pilot-testing phase also suggested that using the LwR:DST enhanced the delivery of care by improving the HCPs’ clinical thinking and communication and increasing their competence, confidence and comfort with the decision-making involved in assessing risk and managing safety concerns. Our data from the pilot-testing phase also suggested that the LwR:DST improves the healthcare experience and outcomes for older adults as they reported feeling heard and involved in these care decisions.

More specifically, our results from both the validation and pilot-testing phases revealed that HCPs from a variety of clinical settings consistently saw the value of the LwR:DST as a tool that enhanced clinical decision-making, broadened their risk assessment process, improved communication and supported different perspectives. These strengths are in line with recommendations for a better discharge planning experience for older adults [30] and are considered essential to the discharge planning process [31–33]. These positive outcomes also align with the known benefits of using decision support tools, such as improving clinicians’ communication of risk [34], reducing decisional conflict [35] and improving elicitation of patient values [36]. The HCPs involved in the pilot-testing noted that the LwR:DST’s process was helpful in making explicit what they did implicitly and also ensured a more consistent and comprehensive risk assessment. In addition, the results suggest that the LwR:DST is flexible enough that it can be used for a variety of concerns in diverse clinical settings. Furthermore, it is innovative in employing worksheets (Additional file 6) that provide visual support to initiate and sustain the dialogue between HCPs, older adults and caregivers in the search for acceptable solutions for safety concerns. The LwR:DST’s content and process also align with elements of the Psycho-Social Rationality Model for risk-managing decision-making proposed by Taylor [37] in that the LwR:DST includes both risk assessment and management, suggests that there is not only one way to reduce possible negative outcomes, encourages the balancing of harms and benefits, supports the discussions concerning alternative solutions, invites the consideration of likelihood and severity, but does not overtly suggest the need for statistics and could be used over time.

The HCPs also provided input regarding how the LwR:DST could be improved. First, they consistently requested more clarity in how to have conversations about risks, especially with people with cognitive impairments. To address the suggestion of having more support in communicating safety concerns, in future iterations of the LwR:DST’s instruction manual we will include a framework for shared decision-making that helps identify the informational needs of older adults and their caregivers [38] as well as a framework for risk communication in dementia care [39, 40]. Second, some HCPs called for more objectivity in the risk analysis step, such as an overall safety prediction number and more guidance on how to quantify the levels in the safety continuum. This request is not surprising as HCPs often feel uncomfortable with uncertainty in complex clinical situations [41]. However, it has been found that safety concerns related to living with risk are fluid and context-dependent [42] and more likely to involve unquantifiable uncertainty [40]. For these reasons, it may not be possible to further objectify the LwR:DST. Lastly, the HCPs also requested clarity on what the tool was and how to integrate it into practice. Calling the LwR:DST a tool induced the HCPs to believe that using the LwR:DST meant completing one of the worksheets. This finding led to a rebranding of the ‘tool’ to the Living with Risk: Decision Support Approach to highlight that this is a 4-step approach to risk assessment and management which does not necessarily require the completion of one of the worksheets. The worksheets were developed to operationalize the approach and participants were invited to use them as needed, especially with initial use. However, the HCPs requested more guidance on when and how to incorporate the worksheets in practice and recommended further improvements in their format. The rebranding should allow more flexibility in LwR:DST use but a current implementation study will address how best to properly balance a formalized yet flexible approach when integrating the LwR:DST into care.

The older adult and caregiver participants in both phases consistently reiterated the importance of being heard, of focusing on their quality of life over safety, of being part of the decision-making process, of knowing their options and of being provided with the rationale for the clinician’s impressions and recommendations. The older adult and caregiver participants also reiterated the importance of having the safe and supportive environment created by the HCPs so that differing views can be brought up and discussed. Our results are in line with previous studies that recommended person-centered care for older adults living with frailty and a shared decision-making approach (including involving the caregiver) when there are safety concerns [30, 43–45]. The patient and caregiver participants’ request to understand the rationale behind the HCPs’ impressions addresses recommendations for older adults to be able to make informed decisions during the discharge planning process [45]. Also, our results describing the difficult experience transitioning care from hospital to home were in line with other studies’ findings [46] as our participants described feeling abandoned by the hospital and requiring medical expertise that exceeded their abilities. Use of the LwR:DST in the pilot-testing phase ensured that the older adult and caregiver were involved in the decision-making process and were provided with the rationale for the HCPs’ impressions and recommendations. Further guidance will be provided in the instruction manual on the importance of supporting caregiver needs during the transition from hospital to home.

This study’s strengths included having over 170 HCPs from different disciplines and multiple settings across Canada provide their input across the two phases of the study. It was also beneficial to have knowledge users on the research team and use research assistants with strong clinical backgrounds. The knowledge users helped provide clinically relevant perspectives throughout the research study and facilitated the recruitment of both HCPs and older adults. Research assistants with over 20 years of clinical experience helped with recruitment and participation of the older adults and caregivers in Phase 2, especially when the interviews had to be done by phone due to public health restrictions. The iterative study design facilitated building on and validating input from knowledge users in each phase, further supporting the evolution of the LwR:DST and ensuring that it remained clinically useful and relevant.

COVID-19 was responsible for some of this study’s limitations. There were delays in starting both phases of the study due to COVID-19, which resulted in a loss of momentum in tool use; also, one site had to retrain for the pilot-testing phase (Phase 2). COVID-19 hampered the ability to recruit older adults for the validation phase (Phase 1), and since the focus groups had to be conducted online due to public health restrictions, this likely resulted in older adults with higher e-literacy being recruited. Due to the burden of COVID-19 on HCPs, recruitment for HCPs and pilot-testing sites could only be done through the research team’s professional contacts, which in turn affected the generalizability of the findings. This limits the transferability of the data regarding how to use the LwR:DST in practice but the positive outcomes highlighted in Phase 1 were consistent with the strengths of the LwR:DST identified in Phase 2. Less input was received on the French version of the LwR:DST due to increased COVID-19 related restrictions regarding accessing healthcare professionals and older adults in this region. When restrictions were eased, an additional 8 HCPs (including a patient representative) were recruited (Table 1) and they answered the Delphi questions on the revised LwR:DST; a patient representative also reviewed the revised LwR:DST and provided feedback. While early findings suggest no difference between the LwR:DST’s two languages, a future study will re-examine whether there are language differences for the LwR:DST.

## Conclusions

Current practice in assessing and managing risk in older adults with complex needs raises issues regarding how to accurately assess and comprehensively manage risks associated with remaining at home in a way that is in line with patient-centered care and can reduce moral distress for HCPs. Using the LwR:DST in practice fills the gap by supporting a more-balanced, systematic, and comprehensive approach to risk assessment and ensures that older adults and their caregivers are involved in the decision-making process related to safety concerns at home. Our findings suggested that the LwR:DST supports a paradigm shift for risk assessment as other risk assessment tools are often less balanced and more prescriptive or have not been adapted to both community and hospital settings [16, 47]. This revised version of the LwR:DST is expected to help hospital- and community-based HCPs accurately and efficiently identify the risks that older adults face when remaining at or returning home and find acceptable ways to mitigate those risks. Challenges remain in integrating the LwR:DST into practice, including when, how and with whom to use it. More work is also required to provide HCPs, older adults and their caregivers with worksheet formats that meet each stakeholder’s needs and can be used in different types of clinical settings. Future research studies evaluating barriers and facilitators when adopting the LWR-DST in real-life settings will address these challenges.

## Supplementary Information

**Additional file 1.**

**Additional file 2.**

**Additional file 3.**

**Additional file 4.**

**Additional file 5.**

**Additional file 6.**

**Additional file 7.**

## Data Availability

The data generated and analyzed during the current study are available from the corresponding author on reasonable request.

## Abbreviations

HCPs: Healthcare professionals
KT: Knowledge translation
LwR: DST:Living with Risk:Decision Support Tool
CHW: Community Health Worker
MD: Medical Doctor
OT: Occupational Therapist
PT: Physiotherapist
RN: Registered Nurse
SW: Social Worker
