# Supplementary material for: Shifting the narrative from living at risk to living with risk: validating and pilot-testing a clinical decision support tool: a mixed methods study
Source: BMC Geriatr. 2023 May 31;23:338. doi: 10.1186/s12877-023-04068-w (PMC10230481; doi:10.1186/s12877-023-04068-w)
Supplement: Supplementary file 1 — Additional file 1. [file 12877_2023_4068_MOESM1_ESM.pdf]

Additional File 1 Delphi Round 1: themes and quotes from participants (health care providers from hospital and community)

Themes	Quotes from participants (setting)
Uses of the tool<br>• Clinical reasoning<br>• Communication<br>• Documentation, Discharge planning	'*I think that it helps to organize ideas and concerns, and facilitates collaborative problem-solving around risk. In my experience, these conversations are already happening but not in a way that the whole team is always involved and there is a clear action plan. I think the tool and the worksheets help to consolidate information and, in the end, save time for everyone because there isn't so much back-and-forth*.' (hospital)<br>'*Excellent tool to guide critical thinking. I appreciate the prompts that encourage the user to consider the client's perspective, not just their own professional thinking*.' (community and hospital)
Strengths of the tool<br>• Comprehensive<br>• Systematic<br>• Patient-centered	'*Risk is extremely subjective and difficult to assess and at times overwhelming at the start. I feel that having a concrete and objective tool will greatly help guide the process of navigating risk mitigation and understanding for the caregiver, patient and professional. I feel this tool will be an excellent resource for initiating challenging discharges and help to get everyone on the same page*.' (hospital)
Areas for improvement<br>• Format: worksheet<br>• Process: how to use, when, with whom<br>• Approach: context is missing, quantitative score<br>• Practical applications: tips for communication, clinical examples	'*I feel like the tool is missing the component of context. Often healthcare professionals like to categorize a clinical risk without being aware of how the client lives and behaves in the community; this is like seeing one piece of a jigsaw puzzle.' (community)*<br>'*It can be lengthy to complete for every client in an acute care setting. Our caseloads are very heavy and there may need to be some criteria for when it would be appropriate to utilize. To me, it would be best used for times when there is a conflict between what is being recommended by the hospital team and what the client/family want*.' (hospital)
Obstacles to use<br>• Time<br>• Team involvement<br>• Engagement	'*Having the time in my practice to apply it. Although I can already see how great it would be to apply it, with the ongoing demands of the job and the number of patients we are expected to see, it may be difficult to add it to our schedules*.' (community)
