# Supplementary material for: Shifting the narrative from living at risk to living with risk: validating and pilot-testing a clinical decision support tool: a mixed methods study
Source: BMC Geriatr. 2023 May 31;23:338. doi: 10.1186/s12877-023-04068-w (PMC10230481; doi:10.1186/s12877-023-04068-w)
Supplement: Supplementary file 2 — Additional file 2. [file 12877_2023_4068_MOESM2_ESM.pdf]

Additional File 2 Validation of Step 4 'Risk Conversations' by older adults: themes and quotes from participants

Themes	Quotes from participants
Content of risk conversations<br>• Knowing condition<br>• Health professionals' impressions<br>• Rationales for decision<br>• Options	*'But I would want to know all the information that's available, not only about me, but what my options are and what the end result would be.'*<br>*'As a caregiver, I would like to know from the health professionals and the doctor specifically what the risks are exactly as they see them.'*<br>*'I'd really like to know the rationale for why I'm not able to do what I want to do.'*
Process<br>• Collaborative conversations<br>• Inviting solutions<br>• Discussing with the right people<br>• Having the information written down<br>• Supporting quality of life over safety<br>• Who makes the decision<br>• Providing options	*'Yes, I think it's important that it's [a] cooperative endeavor, not the health professional or whoever sits there with a tick sheet. I would probably want to actually see the form and see what we're looking at and see what we're doing. Not be talked at, but more with.'*<br>*'I wouldn't want to be talked down to. I think that's really, really important because if somebody does that, then you just stop listening. So the conversation is on an equal footing.'*<br>*'Well, I think it should be a group effort, in consultation with the doctor, the health professional, the caregiver and the patient. Depending on how the patient is, they should all be consulted and share in the decision-making.'*<br>*'…not a "you can't go home" kind of conversation but [a] "how can I get you home" [conversation].'*<br>*'Sometimes when you're having conversations you miss parts, pieces of it, and so if you have something written down, even if you're the patient, you can look at it and see what your options are and what it may mean.'*<br>*'I'm going to step back into my previous life of having a 98-year-old mother living with me since she was 96 and I really believe that it's important to capture risk versus quality of life, and I really think we need to understand because as these people age and live to be older and older and lose people, they know they're at risk. So maybe the issue becomes the quality and I think that's paramount in helping them to make decisions, too, because if they – I think back to our situation and staying at home was paramount. That was what mattered, no matter what.'*
