# Supplementary material for: Shifting the narrative from living at risk to living with risk: validating and pilot-testing a clinical decision support tool: a mixed methods study
Source: BMC Geriatr. 2023 May 31;23:338. doi: 10.1186/s12877-023-04068-w (PMC10230481; doi:10.1186/s12877-023-04068-w)
Supplement: Supplementary file 3 — Additional file 3. [file 12877_2023_4068_MOESM3_ESM.pdf]

Additional File 3 Pre-training: qualitative data (n=19)

**How do you currently assess risk in your patients?**		
**CONTENT**		
**Impairment level**	Cognition	• Assess cognition
	Physical ability	• Assess physical function<br>• Assess mobility<br>• Assess falls
	Medication	• Assess medication (taking as prescribed, high-risk meds, etc.)
**Environment**	Physical environment	• Assess the physical environment in the home<br>• Assess hazards
	Social environment	• Assess for types of support required<br>• Assess available informal and formal support<br>• Assess quality of relationships
**Function**	Functional abilities	• Use functional assessments
**Concerns**	Risks	• Use risk assessment tool
	Concerns	• Assess for different types of risk... Risk of falls, risk of homelessness, etc.
**PROCESS**		
**Gather data**	Assess	• Gather information from patient and referral source<br>• Use standardized assessments for cognition and function<br>• Use non-standardized assessments (functional)<br>• Find out previous level of function from collateral sources<br>• Use assessment findings from team members' reports that identify risks<br>• Review chart
	Observe	• Use informal observation<br>• Use observations of home environment
**Communicate**	Ask	• Ask patient about safety concerns<br>• Obtain information from families and/or caregivers
	Conversations	• With patient<br>• With family and/or caregivers<br>• With community resources<br>• With team
**How do you currently manage risk in your patients?**		
**CONTENT**		
**Impairment**	Mobility	• Risks are managed with a combination of ensuring the patient is able to ambulate safely and has the proper equipment, educating the patient and developing a physical therapy program specific to their needs

**Environments**	Social environment	• Make recommendations for formal (home care, community resources) and informal support (family assistance)<br>• Recommend devices/compensatory strategies<br>• Educate patients and families<br>• Recommend higher level of care if necessary<br>• Problem-solve types of support available and weigh the risks
**PROCESS**		
**Gather data**	Assess	• Assess comprehension<br>• Assess capacity
	Identify	• Areas they are at risk
**Treatment**	Arrange	• Refer to PT/OT for further risk assessment
	Connect	• Consult with providers for further treatment
	Educate	• Educate patients and caregivers
	Provide solutions	• Provide information for community resources that help mitigate risk
	Mitigate	• Make recommendations to mitigate risk
	Communicate	**Discuss**<br>• Areas of concern and options with multidisciplinary team members<br>• Discuss strategies to mitigate risk<br>**Communicate**<br>• Inform patients about risks and let them decide how to manage their own risks
	Meet	• Early discharge planning
	Document	• Document concerns and recommendations
**Approaches**	Patient centeredness	• Discuss values and beliefs with patients
	Support patient's right to self-determination	• Inform patients about risks and let them decide how to manage their own risks
**What are your current difficulties in assessing and managing risk with your patients?**		
**CONTENT**		
**Impairments**	Cognition<br>Capacity – Poor insight	• Patients' insight<br>• Cognitive impairment and capacity
	Fluctuating abilities	• Always changing
**Environment**	Social environment	**Support**<br>• Reliability of family and community resources<br>• Availability and amount of community resources<br>**Programs**<br>• Wait times for higher levels of care<br>• Availability of home care services

	Hospital environment	• Prediction of future function at home<br>• Impact of hospital environment on performance, COVID-19 related restrictions (family unable to also participate in therapy, isolation after returning home), unable to do as many home visits
**PROCESS**		
**Gather data**	Limited information	• Remember to ask all/enough questions to get the full picture
**Treatment**	Follow-through	• Decreased buy-in from patients and families
**Approaches**	Dissonance (wanting different things)	• Conflicting opinions and perspectives (teams, caregivers)<br>• Inconsistent messaging to patients/families regarding risks and recommendations
	Helplessness	• Keep patient safe at all times
