# Supplementary material for: Shifting the narrative from living at risk to living with risk: validating and pilot-testing a clinical decision support tool: a mixed methods study
Source: BMC Geriatr. 2023 May 31;23:338. doi: 10.1186/s12877-023-04068-w (PMC10230481; doi:10.1186/s12877-023-04068-w)
Supplement: Supplementary file 4 — Additional file 4. [file 12877_2023_4068_MOESM4_ESM.pdf]

Additional File 4 Phase 2 HCP focus groups: themes

Themes	Quotes from participants (setting)
Usefulness<br>• Clinical thinking (identifies, clarifies and/or prioritizes concerns; supports, validates and structures thinking, makes implicit thinking explicit, supports reflection)<br>• Communication (initiates, supports, structures, clarifies, focuses communication and/or provides the rationale)<br>• Outcomes (addresses moral distress, finds solutions, manages risk, and/or supports decision-making)<br>• Process (confirms, evaluates, formalizes, identifies care team, justifies discharge, structures process, supports documentation, supports review/re-assessment, supports continuity of care, clarifies use – by and for whom)	'*I referred back to that a lot when I was using the decision tool because it helped me realize that maybe my concerns aren't as worrisome as they should be. I said: "Maybe it's only a yellow and not a red." Even though I think it's a red, my client thinks it's a green. I think having that color-coded chart is really helpful to use with the tool*.' (community)<br>'*I think for that patient it was making sure that the family and patient were on the same page, understood our concerns, and the impact for the next however long of this patient's life. I think that was helpful and the patient seemed to be very appreciative. And the family as well, they seemed to just understand where we were coming from*.' (hospital)<br>'*With one of my caregivers, I think it helped her feel validated that her concerns are also the concerns of the healthcare professionals and that she has a valid reason why she has those concerns. I think it just made her feel good. At the end when I asked her: "Okay?" She said: "That tool was great"*.' (hospital)<br>'*I can see it being helpful if we use it consistently and just as [colleague] said, it's something that can help you say: "Ok, this is what we identified. This is what we offered. We offered A, B, C, D and it was declined." It's a great tool to know that I'm not just discharging them because I want to get rid of them. These are the reasons why. And just put it in a formal way so they understand. You know what I mean? Because those conversations can be difficult to have and if you have a tool like that, there's no emotion there, it's facts*.' (community)
Fit<br>• Type of patients (in keeping with patients' needs)<br>• Useful when there are different perspectives	'*It was really—at the beginning, to be honest, it was more work for me, but when I chose two of my clients, it was helpful, really. I was surprised because at the beginning, to be honest, [my reaction] was, oh no, oh man. But after working with the client, and my client, one of them specifically, she was pleased with the questionnaire and everything. Yeah, and we're still talking about that. I think it was a great tool.*' (community)<br>'*It was a very good fit because in team rounds, we often have patients who are living at risk or who want to—you know, they're adamant about going home but they have high*

	risk, and we would suggest that [they] move to a more supportive living environment.' (hospital)
Improvements<br>• Format (worksheets, length)<br>• Content (what the tool is, how to use worksheets, how to rate risk)<br>• Process (how to integrate into practice, how to use with patients with cognitive impairments)	'*I found it was a bit cumbersome to go through each question during doctors' rounds, which is normally how we get together to go over each patient. With that being said, I think making the Decision Support Tool a little bit shorter or more condensed would help with the 4-step approach*.' (hospital)<br>'*When I use this tool, I use that Risk Analysis Worksheet as the tool. Was that the right thing to do? Or was I supposed to ask those four questions? I'm a bit confused, I know it's after we've already used the tool. So I'm just wondering, should I have done that, was that right?*' (community)<br>'*And also the chart, the green, red, yellow thing. Like [colleague] said earlier, it would be great if you had an idea, you know how it says if you have this many reds, then it's a red, if you have two reds and two yellows, then this is how you decide what [risk] status you're at. It would be great to have some kind of guidelines because even though you knew this [is] red, this is green, this is green or yellow, at the end you didn't really know what purpose that served. You know what I mean, because it didn't give you a final way of deciding what color that one section would be*.' (community)
Obstacles to use<br>• Integration into current practice<br>• Subjectivity of risk	'*It has to be easy. For us, it'd be easier if everything was on our computer system, [if] it was there, we'd just pull it up, fill it in and then we were done. But because we have so many extra steps, we're less likely to do all these processes because it's extra work for us at that point*.' (community)<br>'*Like right now, it's so separate, I honestly don't even think about it. What do I need to bring to my intake and I don't even think about it. But if it was integrated in the whole process already from the get-go, then it would just be natural to use it and it would be helpful. And, like we discussed having the client be there in person, see it happen, have those options like we just talked about. Then yes, it would definitely be helpful for sure*.' (community)
Impact	'*"Well, what are you going to do with this?" And I said: "I'm doing these to see where your strengths are, where your weaknesses are, and what we can do to help you function at*

• Patient (facilitates improved conversations)<br>• Health professional (improves confidence, validates decision-making, increases clarity of thinking)	*your best, your highest levels. By doing this we're going to give you ideas on how to keep [you] safe, and how to, you know, mitigate or decrease the risk of living at home, etc." He was really happy about that, he said: "Okay, I'll do anything you need me to. Just get me home." So I think that helped, and having him say: "I'll talk to anyone about living at risk, yes, I want to be part of this study!".'* (hospital)<br>'*I think for me, it's made me feel more confident talking about these things and discussing the concerns. Kind of like the script. Scripting helps. It increases confidence to start zeroing in and talking and trying to work through the four-step approach, that kind of thing. It gives you…an education on how to proceed and how to engage the team for this discussion*.' (community)
