# Supplementary material for: Shifting the narrative from living at risk to living with risk: validating and pilot-testing a clinical decision support tool: a mixed methods study
Source: BMC Geriatr. 2023 May 31;23:338. doi: 10.1186/s12877-023-04068-w (PMC10230481; doi:10.1186/s12877-023-04068-w)
Supplement: Supplementary file 5 — Additional file 5. [file 12877_2023_4068_MOESM5_ESM.pdf]

Additional File 5 Pilot-test: Themes and quotes from older adults and caregivers

Themes	Quotes from participants
Satisfaction<br>• Feeling heard<br>• Being engaged	'*I thought they handled things pretty fairly. They listened to what I had to say, and I didn't like—I didn't want them to boss me around, so to speak. They listened to me, and they cooperated with me and so in that respect, I was quite satisfied*.' (hospital older adult participant)<br>'*I think that everything was set up, and all the planning and conversations were excellent. It's just once, kind of, the hand-off occurred, the follow-through needs some work*.' (hospital caregiver participant)
Content<br>• HCPs' interpretations and explanations<br>• Roles	'*Well, [HCP] would remind her how important it was to accept assistance and help so she can stay in her home. And many times my mother brings up that she was a nurse. She knows what she's doing. She doesn't need help and the doctor doesn't know what he's talking about. Stuff like that. So [HCP] was really good at explaining it in a way that she wouldn't get confrontational. And she would accept it. But at the same time she wouldn't accept it if we're trying to get meals or someone to come help and clean, stuff like that, she wouldn't accept it.*' (community caregiver participant)<br>'*I don't have all the terminology or a specific understanding of exactly what the right things to say are—terminology would be a good way of explaining it, and I found myself in awkward territory because I was trying to explain to the homecare leaders: "These are the things we understood were going to happen, but this isn't what's going on, and why? Why aren't you doing what you said you were going to do?*' (hospital caregiver participant)<br>'*Yeah, and if people don't have advocates like myself, if seniors are on their own, I see this could be a big issue and a struggle*.' (hospital caregiver participant)
Process<br>• Transitioning home<br>• Decision-making	'*Again, back to what I understood, and I very clearly understood that these were specific requirements that needed to be followed through on, and they didn't happen. It would have been good for the [hospital], or some other transition coordinator, somebody throughout the health services, or somebody else, to step in and right the ship, rather than having it be me*.' (hospital caregiver participant)

	'*I also talked to the [hospital] and they were supposed to send out an occupational therapist to come and see how I was living, that was two weeks ago. And then the doctor said that because of Covid, they can't send anyone out right now. I don't know whether the occupational therapist is someone I could talk to about stuff like this. I don't know. I just feel very depressed right now and so it's hard to know who to turn to or what to do. Because nothing is the same anymore anyway*.' (community older adult participant)<br>'*She wanted to go home; we thought it was probably time for her to get more help but we felt that it needed to come from someone else, from a health professional. She didn't accept that I had the knowledge or information or skill to be able to make that assessment, so at the end of the day it worked out best because the doctors and nurses were able to explain to her what was going on with her health that needed more attention, and she accepted that*.' (hospital caregiver participant)
Therapeutic relationship<br>• Being understood<br>• Instilling hope<br>• Being positive	'*Well definitely, it's my life and my decision, and I just wanted to make sure that they understood where I was coming from and it went fairly smoothly. Everybody had their input, including me*.' (hospital older adult participant)<br>'*In general, I have a lot of problems that drag me down or make me feel ill, basically because I can't handle them, and then she'll [HCP] give me a little tip on phoning a certain person or something. Or else an answer that doesn't completely depress me. And then you feel you have the guts, that it's not impossible. Then you feel like there is hope that they can solve these problems, you know, one by one. Instead of not solving them at all. So I don't give up*.' (community older adult participant)<br>'*I would mention one more point [Sure!]. In the future, I think that a positive attitude shown by the people involved, including myself, is a big benefit, and any negativity should be set aside and mentioned, but not insisted on, type-of-thing, you know? I think that being positive about the whole situation is quite an asset*.' (community older adult participant)<br>'*Yeah, I mean… I guess my only comment was that, you know, [HCP] really did a great job. She smoothed everything over and maybe some of that was her personality. You know, she's well suited for it, etc. I don't know how much of her effectiveness came from the hospital's procedures versus her own personal ability to communicate and understand and kind of get it, you know what I mean?*' (hospital caregiver participant)
