# Supplementary material for: Shifting the narrative from living at risk to living with risk: validating and pilot-testing a clinical decision support tool: a mixed methods study
Source: BMC Geriatr. 2023 May 31;23:338. doi: 10.1186/s12877-023-04068-w (PMC10230481; doi:10.1186/s12877-023-04068-w)
Supplement: Supplementary file 6 — Additional file 6. [file 12877_2023_4068_MOESM6_ESM.pdf]

Living with Risk : Decision Support Approach

RISK ANALYSIS WORKSHEET #1

At risk of

**Concern**	
**Context(s)**	

WHO is concerned
+ level of risk

☐ Clinician	☐ Older Adult	☐ Caregiver

Cause(s) of the concern

Potential consequence(s) of this concern

Recommendations to reduce the concern, its causes and its consequences	Older adult in agreement: YES	Older adult in agreement: NO	Options/ alternatives
	☐	☐ →	
	☐	☐ →	
	☐	☐ →	

Older adult's perspective

RISK ANALYSIS WORKSHEET #2

At risk of

Concern

Context(s)

WHO is concerned

+ level of risk ● ▼ ■

- [ ] Clinician
- [ ] Older Adult
- [ ] Caregiver

Cause(s) of the concern

Potential consequence(s) of this concern

Recommendations to reduce the concern, its causes and its consequences

Recommendations	Older adult in agreement: YES	Older adult in agreement: NO	Options/ alternatives
	☐	☐ →	
	☐	☐ →	
	☐	☐ →	

Older adult's perspective

Living with Risk: Decision Support Approach

RISK ANALYSIS WORKSHEET #3

At risk of

**Concern**	
**Context(s)**	

WHO is concerned

+ level of risk ● ▼ ■

☐ Clinician	☐ Older Adult	☐ Caregiver

Cause(s) of the concern

Potential consequence(s) of this concern

Recommendations to reduce the concern, its causes and its consequences	Older adult in agreement YES	Older adult in agreement NO	Options/ alternatives
	☐	☐ →	
	☐	☐ →	
	☐	☐ →	

Older adult's perspective

RISK ANALYSIS WORKSHEET #4

At risk of

Concern

Context(s)

WHO is concerned

+ level of risk ● ▼ ■

☐ Clinician	☐ Older Adult	☐ Caregiver

Cause(s) of the concern

Potential consequence(s) of this concern

Recommendations to reduce the concern, its causes and its consequences	Older adult in agreement: YES	Older adult in agreement: NO	Options/ alternatives
	☐	☐ →	
	☐	☐ →	
	☐	☐ →	

Older adult's perspective

RISK ANALYSIS WORKSHEET #5

At risk of

**Concern**	
**Context(s)**	

WHO is concerned
+ level of risk ● ▼ ■

☐ Clinician	☐ Older Adult	☐ Caregiver

Cause(s) of the concern

Potential consequence(s) of this concern

Recommendations to reduce the concern, its causes and its consequences	Older adult in agreement YES	Older adult in agreement NO	Options/ alternatives
	☐	☐ →	
	☐	☐ →	
	☐	☐ →	

Older adult's perspective

Living with Risk: Decision Support Approach
SUMMARY TABLE

What is the older adult at risk of? (concern + context)	Who is concerned? What is the risk status? (low ● /medium ▼ /high ■)[1]			Causes	Consequences	Recommendations to minimize concerns, causes and consequences	Older adult in agreement? (yes/no)	Alternatives
	Clinician	Older adult	Caregiver					

[1] Refer to *Living with Risk: Decision Support Approach*- Safety Continua

LWR: DSA
