# Supplementary material for: Shifting the narrative from living at risk to living with risk: validating and pilot-testing a clinical decision support tool: a mixed methods study
Source: BMC Geriatr. 2023 May 31;23:338. doi: 10.1186/s12877-023-04068-w (PMC10230481; doi:10.1186/s12877-023-04068-w)
Supplement: Supplementary file 7 — Additional file 7. [file 12877_2023_4068_MOESM7_ESM.pdf]

Additional File 7 Questionnaires

Pre-Study Phase: Focus Groups Questions with HCPs, Older Adults, Caregivers, Researchers

1. In what way was this decision support tool different from the one you currently use?
2. What obstacles/limitations (either at an individual or organizational level) do you see in using this tool?
3. What strengths do you see in using this tool?
4. What would be your recommendations to improve the format and the content of the tool (to the hospital environment)?

Phase 1: Delphi Questionnaire with Healthcare Professionals

1. **Round 1**
    a. Demographic
        i. Practice location
        ii. Years of experience
        iii. Discipline
        iv. Province of practice
        v. Language of tool reviewed
    b. To what extent do you perceive the tool as useful to your work in assessing and managing risks that older adults may face (responds to an unfulfilled need, intends to use it, has enough details/sufficient information, reflects what you think should be done)? (1-3=not useful, 4-6= somewhat useful, 7-9=useful)
    c. To what extent do you perceive each step of the tool as useful to your work in assessing and managing risks that older adults may face (1-3=not useful, 4-6= somewhat useful, 7-9=useful)
        i. Step 1: What is the older adult at risk of and who is concerned?
        ii. Step 2: What is the older adult's risk status?
        iii. Step 3: What can be done about the concerns?
        iv. Step 4: How to have a conversation about risks and ways to address concerns?
    d. Does the 4-step approach contain adequate content to be able to assess and manage the risks associated with older adults? (1-3=not adequate, 4-6= somewhat adequate, 7-9=adequate)
    e. To what extent do you perceive each step of the tool contains adequate content to be able to assess and manage the risks associated with older adults? (1-3=not adequate, 4-6= somewhat adequate, 7-9=adequate)
        i. Step 1: What is the older adult at risk of and who is concerned?
        ii. Step 2: What is the older adult's risk status?
        iii. Step 3: What can be done about the concerns?
        iv. Step 4: How to have a conversation about risks and ways to address concerns?

f. If you indicated that the tool or any steps of the tool did not contain adequate content (ratings 1-3), or somewhat adequate content (ratings 4-6) please describe why it was not adequate and what would make the tool more adequate (i.e. what information is missing or what information should be removed).
g. For Step 1 does this list represent the concerns you have about your older adult patients/clients? (Step 1: what is the older adult at risk of and who is concerned?)
    i. Should any concerns be added or removed? If yes, please elaborate.
h. To what extent is the format of the tool 'user-friendly' (organization of the ideas, flow in 4-steps, use of colours, length, etc.)? (1-3=not user-friendly, 4-6= somewhat user-friendly, 7-9= user-friendly)
    i. Please provide any additional feedback on making the format of the tool more 'user-friendly':
i. Is there any terminology used in the tool that needs to be: Clarified? If yes, please provide the terminology to be clarified. Changed? If yes, please provide the terminology to be changed.
j. What obstacles/limitations do you see in using this tool?
k. What strengths do you see in using this tool?
l. Do you see any 'added value' to using it?
m. Do you have any other comments about the tool?
n. Should any of the following sections of the instruction manual be expanded, removed or modified? Background (what is it, how was it developed, the clinical context); Clinical examples of the tool; Foundational supporting concerns; Frequently asked questions; Appendices.
o. Should any information be added or expanded (in the instruction manual)? If yes, please elaborate.
p. Should any information be removed or modified? If yes, please elaborate.
q. To what extent is the format of the instruction manual 'user-friendly' (use of colors, length, organization of the information, etc.)? (1-3=not user-friendly, 4-6= somewhat user-friendly, 7-9= user-friendly)
r. Please provide any additional feedback on making the format of the instruction manual more 'user-friendly'.
s. Is there any terminology used in the instruction manual that needs to be: Clarified? If yes, please provide the terminology to be clarified. Changed? If yes, please provide the terminology to be changed.
t. Do you have any other comments about the instruction manual?

2. Round 2

a. To what extent, do you agree that it is clinically useful for the safety continua to include the colors (1-3=do not agree, 4-6= somewhat agree, 7-9= agree)
b. As we make changes to the format based on your feedback from Delphi 1 to what extent do you agree that it is clinically useful to have a worksheet (page 29 of the instruction manual) that can be filled out (i.e. paper, fillable PDF, online, app, embedded EMR etc.)? (1-3=do not agree, 4-6= somewhat agree, 7-9= agree)
c. Do you have any other comments?

Phase 1: Focus Groups Questions with Older Adults

1. About conversations with clinicians when safety is a concern:
    a. What do you need to know?
    b. How do you want to have these conversations?
    c. What do we need to tell clinicians that is important to you for these conversations?
    d. What is important to you in these conversations?
2. About the Summary Table Worksheet:
    a. Is this summary table helpful?
    b. Is there too much information?
    c. Which columns are helpful?
    d. Which columns would you remove?
    e. Is there anything missing?
    f. Is there any other information that you need?
    g. Would you want a copy of it?
    h. When would you want to see it?
3. Was there anything else that you wanted to share about this health care dilemma (clinicians having conversations with patient and caregivers when safety is a concern) that we did not ask?

Phase 2: Pre-Use Pre-Training Questionnaires with Healthcare Professionals

1. What is your current profession?
2. How many years of work experience do you have as a clinician?
3. How many years of work experience do you have in your current workplace?
4. How do you currently assess risk in your patients?
5. How do you currently manage risk in your patients?
6. What are your current difficulties in assessing and managing risk with your patients?
7. You feel confident in your ability to have conversations about discharge and/or care planning with your complex patients. (1=Strongly disagree – 10=Strongly Agree)
8. You feel informed as to how to assess risk for your patients (1=Strongly disagree – 10=Strongly Agree).

Phase 2: Pre-Use Post-Training Questionnaires with Healthcare Professionals

1. Did the workshop provide you with resources and/or strategies to enhance your clinical practice for patients living with risk? (1=Strongly disagree – 10=Strongly Agree)
2. You feel confident in your ability to have conversations about discharge and/or care planning with your complex patients. (1=Strongly disagree – 10=Strongly Agree)

3. You feel informed as to how to assess risk for your patients (1=Strongly disagree – 10=Strongly Agree).
4. Please provide any feedback on this training session.

Phase 2: Post-Use Focus Group Questions with Healthcare Professionals

1. What is your current profession?
2. How many years of work experience do you have as a clinician?
3. How many years of work experience do you have in your current workplace?
4. To what extent did the LwR:DST Fit (aligns with goals of care – content and processes of care – logistics) with clinical needs and constraints with your clinical context?
    a. How did it fit?
    b. How did it not fit?
5. What is your perception on the usefulness of the LwR:DST in decision-making around seniors' safety and autonomy at home?
    a. In what way(s) was it useful?
        i. With which clients was it useful (profile, concerns)?
        ii. In what part of the client journey was the tool useful?
    b. What part(s) of the tool was useful/not useful?
        i. The 4 steps?
        ii. Worksheets?
        iii. Language or wording?
    c. Was it useful to use as a clinician or a team?
    d. Was it useful to discuss with colleagues?
    e. What impact did it have for you? (ie. improved sense of competency re assessment or management?)
    f. What impact do you think it had for the client? (ie. perception of their decision making, understanding of risks/recommendations)
    g. In what way was it not useful?
    h. In what way can we make improvements (any suggestions for improvements?)
6. Would you see any value in using a fillable format of the Risk Analysis Worksheet?
7. Was there anything else that you wanted to share about the tool that we did not ask?

Phase 2: Post-Use Interview Questions with Older Adults and (Caregivers)

1. What is your year of birth and age?
2. What is your primary language?
3. Do you live alone? If not, who do you live with?
4. What was the main topic of conversation or focus of the appointment that you (your older adult) had with Clinician X on Date Y (community)? What was the main topic of conversation you (your older adult) had with your team before you left the hospital?
5. Did you feel that your (older adult) care goals were met? If not, what were you hoping that this conversation/appointment would have achieved?
6. What was the outcome of the conversation/appointment?

7. Did you feel involved in this conversation? If anything other than yes, what would have made you feel more involved?
8. Did you feel heard? If anything other than yes, what would have made you feel more heard?
9. Did you feel that you were given a chance to provide your perspective?
10. Was there any information that you would have liked to have provided? If yes, please describe what you have liked to share?
11. Was your caregiver involved with this conversation? If yes or no, how did you feel about this?
12. Do you have any suggestions on how the conversation could have been improved?
13. Do you have any safety concerns (about your older adult) with living at home? If yes, what concerns do you have? What are you doing (have you put anything in place) to address these concerns? Are you satisfied with how you (your older adult) address these concerns? Have you talked to your (older adult's) clinician about these concerns?
14. Do you (your older adult) avoid doing any day-to-day activities because of any safety concerns? If yes, what activities do you (your older adult) not do? Why did you (your older adult) stop? What are you concerned about?
15. Does your caregiver(s) have any concerns about your safety? If yes, what are they concerned about? Do you agree with their concerns?
16. Did clinician X/team discuss any safety concerns with you? If yes, what where they concerned about? Do you agree with their concerns?
17. Were there any other comments that you wanted to share about your conversation/appointment with clinician/team?
